# Supplementary material for: MetaRibo-Seq measures translation in microbiomes
Source: Nat Commun. 2020 Jun 29;11:3268. doi: 10.1038/s41467-020-17081-z (PMC7324362; doi:10.1038/s41467-020-17081-z)
Supplement: Supplementary file 10 — Supplementary Data 7 [file 41467_2020_17081_MOESM10_ESM.zip › File2/Confidence_High_Taxonomy/89900_out.krona.html]

Javascript must be enabled to view this page.

members
magnitude
magnitudeUnassigned
count
unassigned
taxon
rank

89900\_out

6

6
superkingdom
2

phylum
2
976

200643
2
class

2
order
171549

171552
2
family

838
genus
2

2

SRS143529\_contig\_number\_33455SRS144701\_contig\_number\_30715
species
28131

phylum
4
256845

1313211
class
4

2053569
4

SRS018836\_contig\_number\_22002SRS065504\_contig\_number\_15281SRS078242\_contig\_number\_31257SRS143895\_contig\_number\_35325
species
